# Expression and Clinical Relevance of SOX9 in Gastric Cancer

**DOI:** 10.1155/2019/8267021

**Published:** 2019-06-02

**Authors:** Patrícia Mesquita, Ana F. Freire, Nair Lopes, Rosa Gomes, Daniela Azevedo, Rita Barros, Bruno Pereira, Bruno Cavadas, Helena Pópulo, Paula Boaventura, Leonor David, Luísa Pereira, Raquel Almeida

**Affiliations:** ^1^i3S-Instituto de Investigação e Inovação em Saúde, University of Porto, 4200-135 Porto, Portugal; ^2^Institute of Molecular Pathology and Immunology of the University of Porto (IPATIMUP), 4200-465 Porto, Portugal; ^3^Institute for Cancer Research, Norwegian Radium Hospital, Oslo, Norway; ^4^Serviço de Oncologia Médica do Centro Hospitalar de Trás-os-Montes e Alto Douro, Vila Real, Portugal; ^5^Faculty of Medicine of the University of Porto, 4200-319 Porto, Portugal; ^6^Biology Department, Faculty of Sciences of the University of Porto, 4169-007 Porto, Portugal

## Abstract

Gastric cancer is one of the most frequent tumours and the third leading cause of cancer-related death worldwide. The investigation of new biomarkers that can predict patient outcome more accurately and allow better treatment and follow-up decisions is of crucial importance. SOX9 (sex-determining region Y (SRY)-box 9) is a regulator of cell fate decisions in embryogenesis and adulthood. Here, we sought to ascertain the relevance of SOX9 transcription factor as a prognostic marker in gastric cancer. SOX9 expression was analyzed by immunohistochemistry in 333 gastric adenocarcinoma cases, and its association with clinicopathological and follow-up data was evaluated. SOX9 nuclear expression was absent in 17% of gastric cancer cases and predicted worse disease-free survival (*P* = 0.03). SOX9 expression was associated with lower risk of relapse in Cox univariable analysis (HR = 0.58; 95% CI = 0.35-0.97; *P* = 0.04). The prognostic value of SOX9 was more pronounced in tumours with expansive growth (*P* = 0.01) or with venous invasion (*P* = 0.02). Two validation cohorts from the Cancer Genome Atlas (TCGA) and the Asian Cancer Research Group (ACRG) confirmed that low SOX9 expression was significantly associated with poor patient outcome. In conclusion, we have identified SOX9 as a biomarker of disease relapse in gastric cancer patients. Further experiments are needed to elucidate its biological relevance at the cellular level.

## 1. Introduction

Gastric cancer is the fifth most common cause of cancer and the third leading cause of cancer deaths worldwide, with more than 700,000 deaths annually [1]. Over the last decades, there has been a persistent and steady decline in incidence and mortality [2], but despite all the progress in diagnosis and treatment of gastric cancer, patient prognosis remains poor, with a 5-year survival of only 25% in Europe [3].

TNM classification remains the major prognostic factor used clinically; thus, TNM staging at diagnosis directly affects the treatment received by patients. However, gastric cancer presents a great heterogeneity in terms of tumour aggressiveness, even within the same TNM stage. For that reason, novel prognostic biomarkers are urgently needed to distinguish different tumour biological behaviours, namely, chemotherapy response, regardless of tumour extension or tumour type [4].

SOX9 is a transcription factor that belongs to the SOX (SRY-related high-mobility group (HMG) box) family, and it is known as a key regulator of developmental processes such as male sex determination, chondrogenesis, neurogenesis, and neural crest development (reviewed in [5]). Germline SOX9 heterozygous inactivating mutations result in campomelic dysplasia, a syndrome characterized by skeletal malformations and central nervous system dysfunction together with abnormalities in other organs, which is frequently associated with XY sex reversal [6]. SOX9 plays a crucial role in the regulation of cell fate decisions and stem cell maintenance during embryonic development and adulthood, also in the gastrointestinal tract [7, 8].

SOX9 was extensively studied in the intestine, where it was shown to be expressed in Paneth cells and in the highly proliferative epithelial cells located at the bottom of the crypts, and to be regulated by the Wnt/*β*-catenin signalling pathway [9]. Additionally, SOX9 was shown to inhibit this oncogenic pathway [7, 10, 11], possibly through a direct interaction with *β*-catenin [12]. SOX9 conditional knockout in the mouse intestinal epithelium resulted in increased proliferation, causing intestinal hyperplasia, and decreased differentiation of Paneth and goblet cells [7, 13]. The observation of multiple microadenomas in these mice was explained by the loss of SOX9 negative feedback on the activity of the Wnt/*β*-catenin signalling.

Several lines of evidence suggest a tumour suppressor activity of SOX9 in the intestinal epithelium. SOX9 overexpression in colorectal cancer cells is sufficient to inhibit cell proliferation [10, 14] whereas SOX9 knockdown increases the proliferation of the human colorectal cancer cells [14]. Moreover, SOX9 levels were shown recently to be inversely correlated with the risk of relapse in stage II colorectal carcinomas [15]. The observations that SOX9 inhibits the expression of oncogenes [16] and stimulates the expression of tumour suppressor genes [14], together with the fact that it is frequently mutated in colorectal carcinomas and cell lines [17, 18], suggest that SOX9 may play a tumour-suppressive role.

However, the role of SOX9 as a tumour suppressor gene in the intestinal epithelium was questioned by some authors. SOX9 knockdown resulted in decrease of proliferation and tumour growth capacity of colorectal cancer cells subcutaneously grafted [19] or injected in the peritoneum of nude mice [20]. Conversely, the overexpression of SOX9 was reported to increase the tumourigenic potential of colorectal cancer cells grafted in nude mice [21]. Regarding clinical value, there are reports correlating high levels of SOX9 with poor prognosis in colorectal cancer [22] and one study enrolling a large number of patients that shows no association with prognosis [23].

In the stomach, SOX9 is less studied. It is expressed in normal gastric mucosa, intestinal metaplasia, and gastric carcinoma [24–26]. Several studies have reported high levels of SOX9 in gastric cancer [25–29], but the association with patient prognosis is poorly defined. One study reported a significant association between high SOX9 expression, advanced TNM stages, and lower overall survival [30], further reviewed in [31]. On the other hand, Sun *et al.* [28] reported that SOX9 expression was decreased in tumours due to promoter methylation and inversely related to the advanced tumour stage, vessel infiltration, and nodal metastasis, but did not associate with patient overall survival.

Our specific aims were to assess the value of SOX9 expression as a prognostic marker using two clearly defined outcomes—patient overall survival and time to relapse—and as a predictive marker of response to therapy in gastric cancer patients. For that, we studied SOX9 expression by immunohistochemistry in a consecutive, single-hospital patient series of primary gastric carcinomas.

## 2. Material and Methods

### 2.1. Patients and Tumour Tissue Samples

This retrospective study includes consecutive gastric adenocarcinoma cases surgically treated between January 2008 and December 2014, at Centro Hospitalar São João, Porto, Portugal, for whom clinicopathological and treatment data, follow-up information, and tumour tissue (*n* = 333) were available. All samples are included in the biobank at Centro Hospitalar S. João and have written informed consent from the patients. The study was approved by the ethics committee at Centro Hospitalar S. João (Ethics Committee references CES 122/15 and CES 117/18). Relevant clinical information on the series is provided in Table 1.

The Cancer Genome Atlas (TCGA; https://cancergenome.nih.gov/) and the Asian Cancer Research Group (ACRG; [32]) datasets of gastric carcinomas were used for validation. The TCGA database was designed by a collaboration between the National Cancer Institute (NCI) and the National Human Genome Research Institute (NHGRI) which is aimed at generating comprehensive, multidimensional maps of the key genomic changes in 33 types of cancer. This database includes careful clinical information and several omics results. TCGA reported 354 cases of gastric carcinomas for which expression data for SOX9 (based on RNA-seq) and updated survival information was available. These 354 cases were from individuals with diverse geographical origins (America, Europe, Asia, and unknown), mean age of 65.54 years old, 35.5% females, and 64.7% males. The ACRG cohort included 300 cases characterized for microarray-based transcriptome (Affymetrix GeneChip Human Genome U133 Plus 2.0; data downloaded from NCBI GEO with identifier GSE62254). These cases were from Asian ancestry, with mean age of 61.94 years old, 33.7% females, and 66.3% males.

### 2.2. Immunohistochemistry

Cores from the available formalin-fixed paraffin-embedded tumour tissues were included in tissue microarrays (TMAs). Sections of 4 *μ*m from the formalin-fixed paraffin-embedded TMAs were obtained for the immunohistochemical study of SOX9 expression. First, tissues were deparaffinised and hydrated. Heat-induced epitope retrieval was carried out in an IHC-Tek Epitope Retrieval Steamer Set for 40 minutes in 10 mM Tris-EDTA pH 9.0. Endogenous peroxidase activity was blocked with 3% hydrogen peroxide for 10 minutes. Primary antibody (anti-SOX9 1 : 6000 dilution, AB5535, Millipore, Merck Group, Darmstadt, Germany) was incubated overnight at 4°C. Detection was done using the Dako REAL Envision Detection System Peroxidase/DAB+ (Dako, Glostrup, Denmark). Sections were then counterstained with hematoxylin, dehydrated, and mounted. Samples were considered positive when >5% of the malignant cells were stained and in consensus of 3 observers.

### 2.3. Statistical Analysis

This study followed the REMARK guidelines to report biomarkers (Supplementary Table 1) [33]. In order to assess the significance (*P* values) of differences in clinicopathological features across the two groups of SOX9 expression in our cohort, we have used different statistical tests. The Student *t*-test was used when comparing with age. Fisher's exact test (2-sided) was used for gender, growth pattern, resection margins, vascular invasion, and perineural invasion, and chi-square (*χ* ^2^) test was used for Laurén classification, WHO classification, and TNM staging. The Kaplan-Meier method was used to generate 5-year disease-free survival (DFS) and overall survival (OS) plots, and its significance was assessed by the log-rank test. DFS was defined as the time from operation to the first recurrence event. OS was defined as the time from operation to death from any cause. Cox proportional hazards model was used to calculate univariable and multivariable hazard ratios (HRs) and confidence intervals (CIs) for disease recurrence. The clinicopathological parameters included in the multivariable models were selected based on their individual clinical relevance and *a priori* knowledge in a full model approach with no other predictor selection. Patients with missing data were not included in the analyses. Differences were considered statistically significant when *P* value < 0.05. Statistical analysis was performed in IBM SPSS Statistics version 24. The expression data inferred by TCGA consortium was used, in fragments per kilobase million (FPKM) units. The highest value (FPKM = 51.7) was used in the density plot of *SOX9* expression to establish the low-expression and high-expression groups and to obtain the Kaplan-Meier OS plots in R (survival and survminer packages) for the entire cohort. Gene expression in the ACRG cohort was inferred on the array raw data provided in GEO, using the RMA (Robust Multiarray Average) methodology for background subtraction and normalization of probe intensities available at the oligo R package [34, 35]. Probe set annotation was downloaded from Affymetrix's website. Two probes mapped to *SOX9* gene, and their average value per sample was used to represent the gene expression. Using the same strategy to establish the low-expression and high-expression *SOX9* groups (threshold = 9.79), the overall survival and disease-free survival were analyzed in the same way as the TCGA cohort.

## 3. Results

### 3.1. SOX9 Expression and Association with Clinicopathological Features in Gastric Carcinomas

Clinicopathological features of the 333 gastric cancer cases and the association with SOX9 expression profiles are summarized in Table 1.

**Figure 1:**
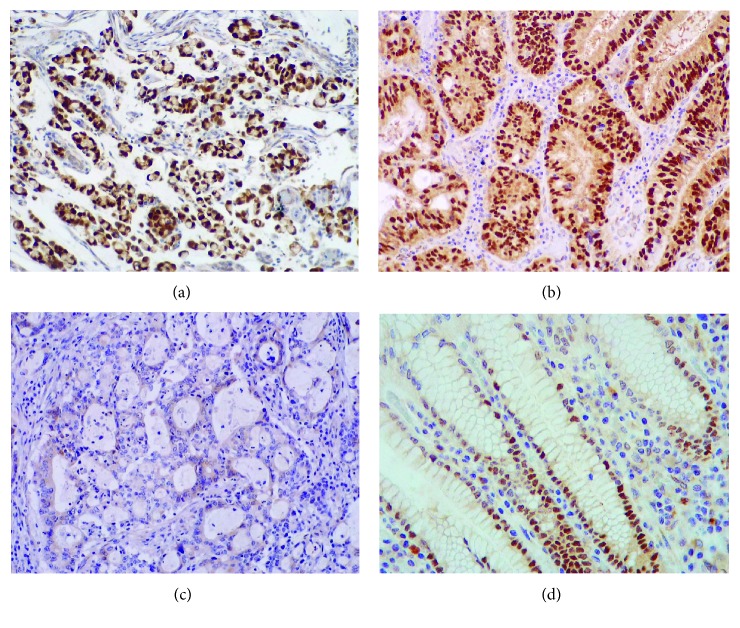
Nuclear expression of SOX9 protein detected by immunohistochemistry (20x magnification). 83% of gastric carcinomas (a and b) express SOX9 in the nucleus. There is a loss of SOX9 expression in 17% of gastric carcinoma cases (c). Nuclear SOX9 expression was also observed in normal gastric mucosa (d).

In 83% (275/333) of the gastric carcinoma cases (Figures 1(a) and 1(b)), SOX9 was located in the nucleus and frequently showed a strong staining pattern. Absence of SOX9 expression was observed in 17% of gastric carcinoma cases (Figure 1(c)). Nuclear SOX9 expression was also observed in normal gastric mucosa (Figure 1(d)), particularly in the neck/isthmus region, as previously described [24, 27].

SOX9 expression was significantly associated with the TNM stage (*P* = 0.04) (Table 1). In stage IV, 92% (60/65) of the patients expressed SOX9. The other clinicopathological parameters, which included age at diagnosis, gender, Láuren classification, Ming classification (growth pattern), WHO classification, tumour clearance at resection margins, vascular invasion, and perineural invasion, were not significantly associated with SOX9 expression. Since SOX9 is a target of the Wnt pathway, we also assessed its association with *β*-catenin expression in different subcellular compartments. Although there was a trend for an increased frequency of gastric cancer samples expressing SOX9 when *β*-catenin was expressed in the nucleus, this difference was not statistically significant (Table 1). Abnormal expression of *β*-catenin in the nucleus, observed in 91/310 (29.4%) of the tumours analyzed, had no prognostic value in this series (data not shown).

### 3.2. Survival Analysis Related to SOX9 Expression

**Figure 2:**
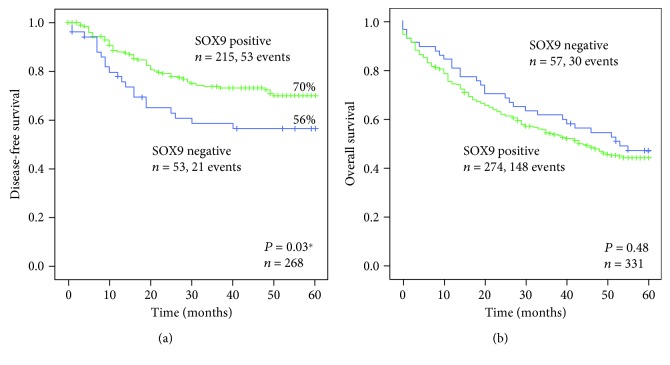
Kaplan-Meier curves showing the probability of (a) disease-free and (b) overall survivals in our series of patients with gastric cancer, according to SOX9 expression.

In order to evaluate whether SOX9 expression in gastric cancer correlated with the patient outcome, Kaplan-Meier curves were constructed, using two clearly defined end-points: time to relapse (disease-free survival) and overall survival. SOX9 expression in the tumours was significantly associated with better disease-free survival (*P* = 0.03), stratifying the patients into two prognostic groups characterized by a 5-year disease-free survival of 70% for patients with SOX9-positive tumours *versus* 56% for patients with SOX9-negative tumours (Figure 2(a)).

This correlation was also observed using the univariable Cox regression analysis for disease-free survival (HR = 0.58; 95% CI = 0.35-0.97; *P* = 0.04; Table 2). No correlation was observed in the multivariable analysis (Table 2). SOX9 expression did not predict overall survival (Figure 2(b)).

**Figure 3:**
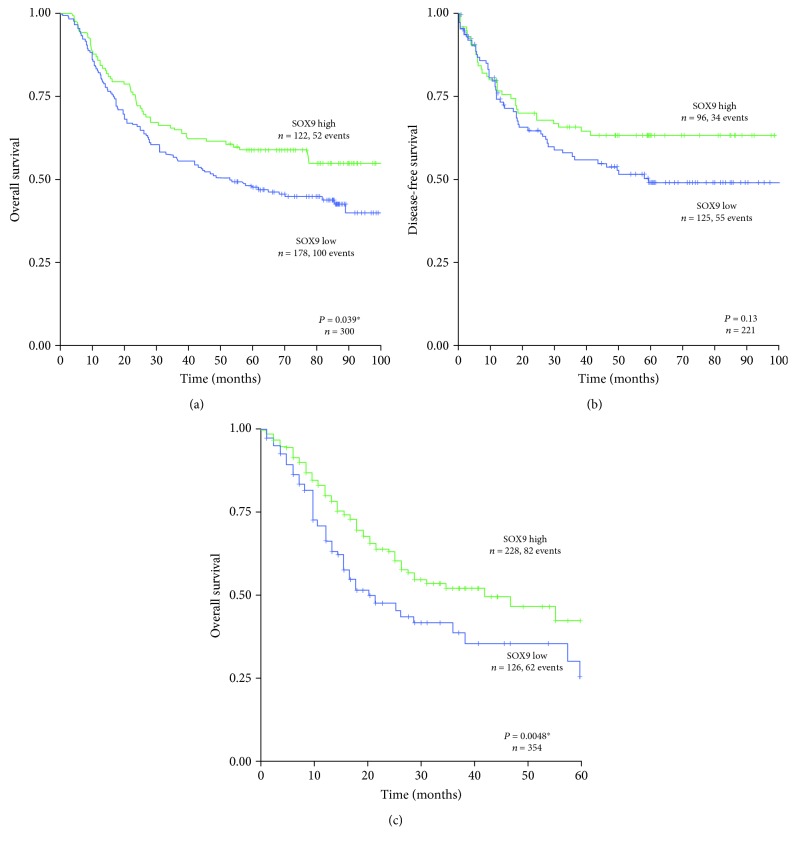
Kaplan-Meier curves showing the probability of (a) overall and (b) disease-free survivals in a gastric cancer validation cohort from the Asian Cancer Research Group (ACRG) series of patients and (c) overall survival in a second gastric cancer validation cohort from the Cancer Genome Atlas (TCGA), all showing high *versus* low *SOX9* expression. ^∗^ *P* < 0.05.

In order to validate our findings in independent cohorts, we analyzed 300 patients with gastric cancer deposited in the Asian Cancer Research Group (ACRG) database and 354 patients deposited in the Cancer Genome Atlas (TCGA) database, for which expression data for SOX9 and updated survival information was available. In these cohorts, low *SOX9* expression levels were significantly associated with decreased overall survival (Figures 3(a) and 3(c)). For the ACRG, patient's disease-free survival data was available and was tested. The same trend was observed (Figure 3(b)), but the association was not statistically significant.

**Figure 4:**
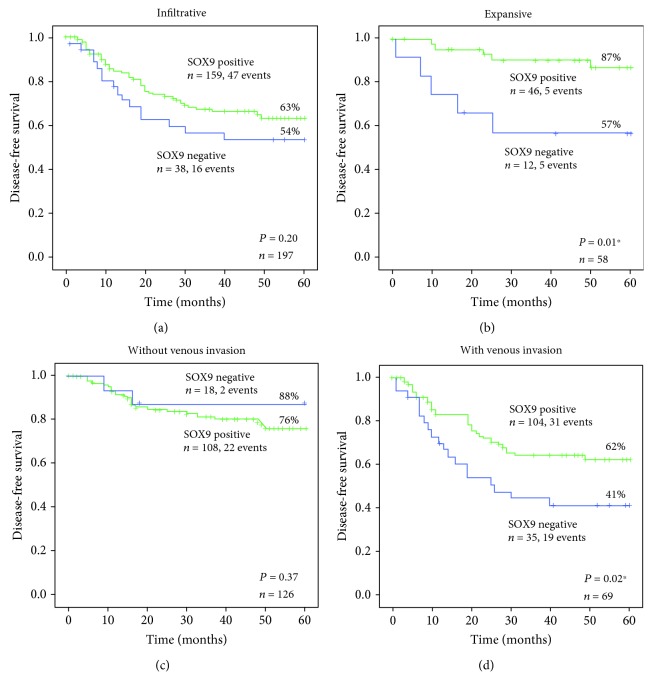
Kaplan-Meier curves showing the probability of disease-free survival for a series of patients with gastric cancer, according to SOX9 expression and (a, b) different growth patterns according to Ming classification; (c, d) occurrence of venous invasion. (a) Infiltrative *versus* (b) expansive growth. (c) Absence *versus* (d) presence of venous invasion. ^∗^ *P* < 0.05.

Then, we assessed in our series whether the SOX9 prognostic value was associated with clinicopathological features. We observed that the SOX9 prognostic value was restricted to tumours with the expansive growth pattern versus the infiltrative growth pattern, according to Ming classification (Figures 4(a) and 4(b)).

In expansive tumours, the 5-year disease-free survival of patients with SOX9-positive tumours was 87% versus 57% of patients with SOX9-negative tumours (*P* = 0.01). Likewise, within cases with venous invasion, the 5-year disease-free survival of patients with SOX9-positive tumours was 62% versus 41% of patients with SOX9-negative tumours (*P* = 0.02) (Figures 4(c) and 4(d)). In fact, combination of SOX9-negative expression and presence of venous invasion defined the group of patients with the worst outcome regarding disease relapse. In cases without venous invasion, there was no difference in disease-free survival according to SOX9 expression. For the other clinicopathological features, SOX9 expression did not define different outcomes regarding disease relapse.

### 3.3. Survival Analysis Related to SOX9 Expression and Response to Chemotherapy

**Figure 5:**
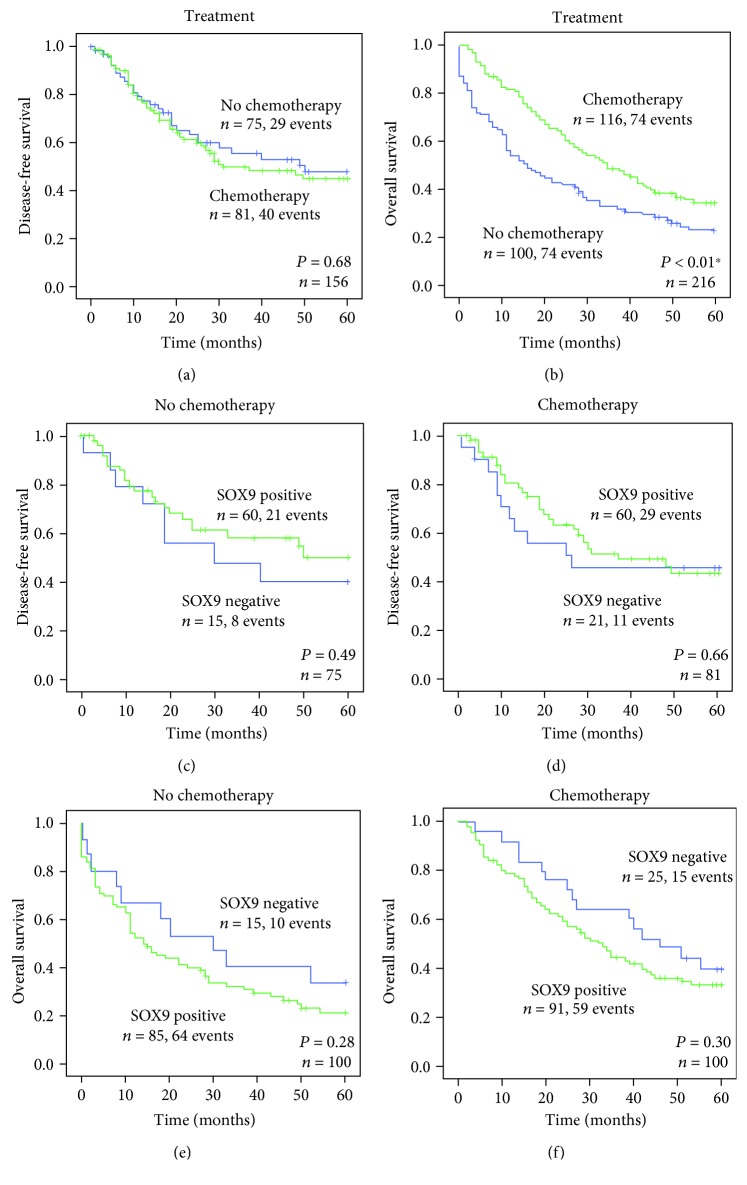
Kaplan-Meier curves showing the probability of (a) disease-free and (b) overall survivals for a series of patients with gastric cancer, according to different treatment options. Stage I tumours were excluded since only 8 patients received chemotherapy. Kaplan-Meier curves showing the probability of (c, d) disease-free and (e, f) overall survivals according to SOX9 expression in (c, e) patients not treated or (d, f) treated with chemotherapy, respectively.

Finally, we assessed whether SOX9 expression was predictive of therapy response in gastric cancer patients. We first assessed the impact of administering chemotherapy, in addition to surgery, in patient disease-free survival and overall survival. Surprisingly, chemotherapy did not significantly improve patient disease-free survival (Figure 5(a)) but improved patient overall survival (Figure 5(b)). In both cases, SOX9 expression did not allow predicting which patients respond better to chemotherapy (Figures 5(c)–5(f)).

## 4. Discussion

In the present study, we use TMA technology to evaluate the clinical significance of SOX9 expression in a large consecutive series of primary gastric cancer from Northern Portugal. We find nuclear expression of SOX9 in 83% of the gastric carcinoma cases from a Portuguese cohort of 333 patients with both clinicopathological and survival data. In addition, we show that the absence of SOX9 expression in the tumour is associated with increased risk of relapse in gastric cancer patients.

The frequency of SOX9 expression observed in this series is higher than the one observed in other reports [27, 28] but close to the one reported by Santos *et al.* [30], in a series of 76 gastric carcinomas from a Brazilian population. The association of SOX9 expression and patient outcome has been addressed in few studies, and the results are not consensual. Most studies [27, 28, 36] did not find a significant association between SOX9 expression and overall survival but did not evaluate disease-free survival. Santos *et al.* [30] found a correlation between a high expression of SOX9 and a poor prognosis based on the ACRG database. On the contrary, the analysis we performed in both the ACRG and the TCGA databases reinforced the worse prognosis associated with low *SOX9* expression in gastric cancer. Although we do not have an explanation for this finding, we speculate that the different results must reside in the cutoffs used to determine SOX9 subgroups. The limitations of our study are related to the shortcomings of using immunohistochemistry protein analyses, which might be affected by tumour heterogeneity and subjective scoring systems. Despite possible limitations, our results are based in one of the largest tumour series used so far to assess the clinical relevance of SOX9 in gastric cancer and the more complete one regarding clinicopathological features, survival data, and treatment data. In addition, we used a simple and easily reproducible scoring system and two validation series. Our results are also in accordance with a study performed in colorectal cancer reporting that loss of SOX9 expression in the invasive front of the tumours predicts tumour relapse in stage II colon cancer patients [15], although the prognostic value of SOX9 is also not consensual in colorectal cancer [22, 23].

*In vitro* studies demonstrated malignant properties of high levels of SOX9 in gastric cancer cells [30, 37–40]. The apparent contradiction between results obtained *in vitro* and the prognostic value we determined in our study suggests a complex role of SOX9 in gastric cancer that might be context and dose-dependent. Loss of SOX9 expression might be more relevant in early-stage disease, which is difficult to assess in cell lines as they accumulate mutations and genomic alterations, hence being more close to advanced tumour stages. On the other hand, SOX9 might have a dose-dependent effect, which has already been suggested for colorectal cancer, where the same apparently contradictory observations have been reported [41]. SOX9 tumour suppressor activity could be due to the modulation of the Wnt/*β*-catenin signalling pathway. It is well established that SOX9 is not only a direct target gene of the Wnt/*β*-catenin pathway but also an inhibitor, having a role in the regulation of epithelial homeostasis [7, 9]. SOX9 levels regulate the transcriptional activity of the *β*-catenin/TCF4 complex, apparently through physical interaction with *β*-catenin, resulting in a competition between SOX9 and TCF4 for binding to *β*-catenin [12]. Formation of the SOX9/*β*-catenin complex results in the degradation of the two proteins and consequently downregulation of the Wnt signalling. Thus, in the absence of SOX9, caused by methylation as described by Sun *et al.* [28] or mutations [17, 18] (reviewed in [41]), the negative feedback regulation of the Wnt-pathway may be lost.

We suggest that SOX9 might be used as a prognostic marker of tumour relapse in gastric cancer patients, which is very important at the clinical level due to the extent of relapse in this type of tumour, even in early stage disease. Furthermore, we show that administering adjuvant chemotherapy to stage II and III patients does not significantly improve the time to relapse, which suggests that the treatment available to gastric cancer patients needs improvement. In this context, the identification of SOX9 as a biomarker of relapse is even more relevant, as it allows the identification of a group of high-risk patients, those with no expression of SOX9 and with signs of venous invasion, that might benefit from a different surveillance scheme and different treatment options, and that need to be studied in more detail.

## 5. Conclusions

In conclusion, this is the first study to report that the absence of SOX9 protein expression in gastric tumours predicts a significant risk of relapse. We anticipate that SOX9 expression could be an important biomarker for the prediction of relapse in gastric cancer stratifying patients to different surveillance schemes or treatment options.

## Figures and Tables

**Table 1:** Clinicopathological data and association with SOX9 in all patients included in the study.

	All cases		SOX9 negative		SOX9 positive		*P*
	*n*	%	*n*	%	*n*	%	
Patients	**333**	100	**58**	17.4	**275**	82.6	
Age							
Media ± SD	**67.6±11.9**		**67.3±9.6**		**67.7±12.4**		0.74
Range	**32-95**		**46-83**		**32-95**		
Gender							
Female	**145**	43.5	**23**	15.9	**122**	84.1	0.56
Male	**188**	56.5	**35**	18.6	**153**	81.4	
Laurén classification							
Intestinal	**156**	46.9	**26**	16.7	**130**	83.3	0.85
Diffuse	**44**	13.2	**8**	18.2	**36**	81.8	
Mixed	**87**	26.1	**17**	19.5	**70**	80.5	
Unclassified	46	13.8					
Growth pattern							
Expansive	**61**	18.3	**13**	21.3	**48**	78.7	0.34
Infiltrative	**258**	77.5	**41**	15.9	**217**	84.1	
Unclassified	14	4.2					
WHO classification							
Tubular	**146**	43.8	**25**	17.1	**121**	82.9	0.99
Papillary	**1**	0.3	**0**	0.0	**1**	100.0	
Poorly cohesive	**37**	11.1	**7**	18.9	**30**	81.1	
Mucinous	**7**	2.1	**1**	14.3	**6**	85.7	
Other variants	**142**	42.7	**25**	17.6	**117**	82.4	
TNM							
I	**112**	33.7	**17**	15.2	**95**	84.8	0.04^∗^
II	**87**	26.1	**21**	24.1	**66**	75.9	
III	**69**	20.7	**15**	21.7	**54**	78.3	
IV	**65**	19.5	**5**	7.7	**60**	92.3	
Resection margins							
R0	**298**	89.5	**53**	17.8	**245**	82.2	0.48
R1/R2	**34**	10.2	**4**	11.8	**30**	88.2	
ND	1	0.3					
Vascular invasion							
No	**140**	42.0	**23**	16.4	**117**	83.6	0.66
Yes	**190**	57.1	**35**	18.4	**155**	81.6	
ND	3	0.9					
Perineural invasion							
No	**173**	52.0	**33**	19.1	**140**	80.9	0.47
Yes	**159**	47.7	**25**	15.7	**134**	84.3	
ND	1	0.3					
*β*-Catenin nuclear expression							
No	**219**	65.8	**43**	19.6	**176**	80.4	0.07
Yes	**91**	27.3	**10**	11.0	**81**	89.0	
ND	23	6.9					
Chemotherapy							
Yes	**124**	37.2	**27**	46.6	**97**	35.8	0.14
No	**205**	61.6	**31**	53.4	**174**	64.2	
ND	4	1.2					

*Notes*. *P* values (statistical significance threshold < 0.05) were obtained using Student's *t*-test for the continuous variable, Fisher's exact test (2-sided), and chi-square (*χ* ^2^) test for categorical variables. ^∗^Comparisons with *P* < 0.05. ND = not determined; SD = standard deviation.

**Table 2:** Disease-free survival univariable and multivariable Cox regression analysis in gastric cancer.

	Number of events	Univariable analysis			Multivariable analysis		
		HR	95% CI	*P* value	HR	95% CI	*P* value
Laurén classification							
Intestinal	28	1			1		
Diffuse	7	1.17	0.51-2.68	0.71	0.71	0.31-1.66	0.44
Mixed	23	1.78	1.03-3.10	0.04^∗^	1.08	0.58-1.99	0.81
Growth pattern							
Infiltrative	62	1			1		
Expansive	10	0.45	0.23-0.88	0.02^∗^	0.52	0.20-1.37	0.19
TNM							
I	5	1			1		
II	26	8.10	3.11-21.12	<0.01^∗^	9.19	2.64-32.01	<0.01^∗^
III	42	24.91	9.81-63.30	<0.01^∗^	23.40	6.74-81-23	<0.01^∗^
Resection margins							
R0	67	1			1		
R1/R2	5	14.33	5.38-38.19	<0.01^∗^	7.83	2.54-25.02	<0.01^∗^
Vascular invasion							
No	24	1			1		
Yes	49	2.28	1.40-3.72	<0.01^∗^	0.90	0.49-1.65	0.73
Perineural invasion							
No	26	1			1		
Yes	47	3.10	1.92-5.02	<0.01^∗^	0.64	0.35-1.17	0.15
SOX9							
No	21	1			1		
Yes	52	0.58	0.35-0.97	0.04^∗^	0.90	0.50-1.62	0.72

*Notes*. *P* values (statistical significance threshold < 0.05) were obtained using univariable and multivariable Cox proportional hazards regression analysis (Wald). ^∗^Comparisons with *P* < 0.05. HR = hazard ratio; CI = confidence interval.

## Data Availability

The data used to support the findings of this study are included within the article.

## Supplementary Materials

Supplementary Materials Table S1: REMARK checklist.
